# Between duty and despair: the ethical toll of brain drain on Nepalese nurse managers

**DOI:** 10.1186/s12912-025-02878-4

**Published:** 2025-02-28

**Authors:** Animesh Ghimire, Mamata Sharma Neupane

**Affiliations:** 1 Research Fellow, Sustainable Prosperity Initiative Nepal, Bhimsengola, Thulo Kharibot, Baneshwor-31, Kathmandu, Nepal; 2 Department of Nursing and Department of Public Health, Chitwan Medical College, Bharatpur-5, Kailashnagar, Chitwan, Nepal

**Keywords:** Brain Drain, Nurse Managers, Ethics, Duty, Despair, Nepal, Healthcare Workforce

## Abstract

**Background:**

The relentless exodus of skilled healthcare professionals from low- and middle-income countries to wealthier nations, known as the 'brain drain,' poses a grave threat to global health equity. Nepal, a prime example of a source country, faces a critical shortage of nurses due to this migration. Nurse managers, uniquely positioned at the intersection of leadership and frontline care, face considerable challenges in times of crisis. These challenges encompass ethical dilemmas associated with resource allocation, staffing shortages, and maintaining quality care standards. Additionally, nurse managers must navigate their own experiences of moral distress, further complicating their decision-making processes and overall effectiveness in managing healthcare teams and patient outcomes. This study delves into their lived experiences, aiming to expose the far-reaching consequences of brain drain and spark a global dialogue on ethical and sustainable healthcare workforce practices.

**Methods:**

This qualitative descriptive study examined the lived experiences of ten nurse managers in Nepal, employing semi-structured interviews and inductive content analysis. Participants were chosen through a combination of purposive, snowball, and convenience sampling methods, ensuring representation from both urban and rural settings.

**Results:**

Thematic analysis revealed five core themes: (1) Moral Distress on the Frontlines; (2) Unequal Burden, Unequal Access; (3) The Ripple Effects of Exodus; (4) Beyond the Hippocratic Oath; and (5) Policy at the Crossroads.

**Conclusion:**

Policy interventions prioritizing improving working conditions, investing in the domestic healthcare workforce, and promoting ethical recruitment practices are essential to ensure equitable and sustainable healthcare. The voices of nurse managers at the forefront of this crisis provide a powerful call to action. Their experiences and insights urge national and global stakeholders to recognize the human cost of brain drain and work collaboratively towards a more just and resilient healthcare system.

**Supplementary Information:**

The online version contains supplementary material available at 10.1186/s12912-025-02878-4.

## Introduction

Brain drain, the migration of skilled healthcare professionals from lower- and middle-income countries (LMICs) to high-income countries (HICs), has garnered significant attention in recent decades. The World Health Organization (WHO) and the International Organization for Migration (IOM) recognize it as a critical challenge to global health equity [1, 2]. The allure of better opportunities, higher salaries, and improved working conditions in developed countries drives many healthcare professionals, including nurses, to seek employment abroad [3]. This departure of skilled labor, often referred to as a "healthcare exodus," [4] has profound implications for the healthcare systems of source countries like Nepal, leading to staff shortages, compromised patient care, and a decline in overall health system performance [5–8]. The ethical dimensions of brain drain have been widely debated, with scholars raising concerns about equity, justice, and the right to health [9]. Marked by one of the highest rates of healthcare worker migration within its region [10], the migration of healthcare professionals from Nepal to developed countries creates disparities in healthcare access and quality, disproportionately affecting vulnerable populations who rely on public health services [11]. Moreover, healthcare professionals face moral dilemmas when choosing between personal career advancement and their duty to serve their home communities [12].

Nurse managers face unique ethical challenges due to their dual roles as leaders and frontline providers. Unlike in many high-income countries (HICs), Nepalese nurse managers are often directly involved in patient care while simultaneously handling administrative responsibilities [13]. This includes tasks such as overseeing ward operations, managing staff schedules, ensuring the availability of essential resources, and directly attending to patients' needs, especially in understaffed conditions. This daily juggling act between administrative duties and hands-on patient care places them at the intersection of organizational expectations and frontline realities, often leading to value conflicts where their professional duties to provide optimal patient care misalign with institutional demands or resource constraints [14]. Such conflicts result in moral distress, a complex psychological phenomenon where nurse leaders recognize the ethically appropriate course of action to uphold patient welfare but feel constrained by organizational limitations, such as inadequate staffing, lack of essential medical supplies, or policies that prioritize cost-cutting over patient well-being [15–17]. Moral distress is not merely a personal emotional response but a significant ethical concern that indicates systemic issues within the healthcare environment [18]. It arises when healthcare professionals are unable to translate their moral choices into action, leading to feelings of powerlessness, frustration, and burnout [19]. In the Nepalese context, the migration of highly skilled nurses further intensifies these constraints. Factors such as low compensation, work-related stress, limited career opportunities, and workplace violence [20], exacerbate the strain on an already overburdened healthcare system [21, 22]. This departure of nurses leads to increased workloads, compromised patient safety, and moral distress among those who remain [23].

While existing studies have examined the broader consequences of brain drain on healthcare systems, including its impact on patient outcomes and workforce shortages [24, 25], there remains a limited understanding of the specific ethical challenges nurse managers encounter in a source country such as Nepal. Nurse managers serving as administrative leaders and direct care providers [8, 26, 27], occupy a unique vantage point from which to observe the multifaceted impacts of brain drain. Their firsthand experience navigating the ethical complexities of staff shortages, compromised patient care, and the emotional toll on the remaining workforce makes their perspectives particularly valuable in understanding the full extent of this crisis [28, 29]. Specifically, these ethical complexities involve dilemmas such as deciding how to allocate scarce resources in an equitable manner, determining the limits of their professional obligations in understaffed conditions, and grappling with the moral distress arising from being unable to provide the desired level of care due to systemic constraints [30, 31]. The insights gained from nurse managers can inform targeted interventions to address the root causes of brain drain, support the well-being of the remaining workforce, and promote ethical and sustainable healthcare practices. To address this critical gap in understanding, this study explores the following question: How do nurse managers in Nepal experience and navigate the ethical implications of brain drain, specifically concerning resource allocation, the limits of their professional obligations in understaffed conditions, and the moral distress they experience, on their professional practice and the healthcare system? The purpose of this study is to explore the lived experiences of Nepalese nurse managers as they grapple with these ethical dilemmas created by brain drain. By amplifying their voices and understanding their challenges, this study aims to inform policy recommendations that promote a more equitable, sustainable, and ethically sound healthcare system in Nepal that values and supports its workforce.

## Methods

### Study design

This study employed a qualitative descriptive design to explore the ethical implications of brain drain on the nursing workforce in Nepal [32], as nurse managers perceive. The study was guided by an interpretive description approach [33] situated within the constructivist paradigm [34]. This paradigm recognizes that knowledge is socially constructed and that reality is interpreted through individual experiences and interactions. In this study, the constructivist paradigm explored the multiple realities and perspectives of nurse managers regarding the ethical implications of brain drain, acknowledging that their experiences are shaped by their unique contexts and interactions within the healthcare system. The study adhered to the Consolidated Criteria for Reporting Qualitative Research (COREQ) guidelines to ensure transparency and rigor in the research process [35].

### Sample and setting

This study was conducted in two hospitals representing diverse healthcare settings within Nepal: an urban hospital in Bagmati Province and a rural hospital in Karnali Province. Bagmati Province is home to Kathmandu, the nation's capital and largest metropolitan area, and represents the most developed and resource-rich region of the country [36]. Conversely, Karnali Province is Nepal's least developed and most geographically remote province, characterized by limited infrastructure, challenging terrain, and significant disparities in access to healthcare services [37, 38]. This geographical isolation is reflected in key development indicators: with 28.9% of its population living below the poverty line and a per capita income of just USD 606 [39], substantially below the national average of USD 1380 [40]. Furthermore, multidimensional poverty affects 51.2% of Karnali's population, and its Human Development Index (HDI) is a mere 0.427, both considerably below the national averages of 28% and 0.49, respectively [39]. These socio-economic disparities translate into significant health challenges, with an average life expectancy of 67 years (the lowest of all provinces), a child malnutrition rate of 58%, and limited access to basic sanitation, with 35.9% of the population lacking access to safe water and only 50% of households having proper toilet facilities [39]. This strategic selection of these two provinces provided a crucial contrast, allowing for the exploration of how brain drain impacts healthcare delivery and the experiences of nurse managers in vastly different contexts within the same country.

The recruitment process for this study aimed to select participants who had a minimum of five years of experience as a nurse manager and could provide rich insights into the ethical implications of brain drain. This selection criterion was deemed appropriate to ensure that participants had sufficient experience navigating the complexities of the healthcare system and witnessing the impact of nurse migration over time. The recruitment process involved a combination of purposive sampling [41], convenience [42], and snowball techniques [43]. Potential participants were initially contacted through invitations distributed by the hospital administrations at each site. These invitations outlined the study's purpose, eligibility criteria, and contact information for the research team. Additionally, flyers with a QR code for self-registration were displayed in prominent locations within the hospitals, allowing for self-selection, which aligns with a convenience sampling approach. Purposive sampling was then used to select participants who volunteered to participate and had direct experience with ethical dilemmas related to nurse migration. In this study, ethical dilemmas refer to situations where nurse managers face difficult choices between competing values or obligations, often arising from the conflict between the needs of individual nurses considering migration and the needs of the healthcare system and the patients it serves. This is distinct from moral distress, which is the psychological distress experienced when one knows the right course of action but is constrained from taking it [44]. While moral distress can be a consequence of ethical dilemmas, the focus here was on identifying participants who had encountered and navigated these challenging situations. To further ensure diverse perspectives within each site (urban/rural), snowball sampling was employed to reach potential participants through existing contacts. While acknowledging the potential for snowball sampling to lead to a homogenous sample, several strategies were implemented to mitigate this risk. Initial contacts were chosen from different departments and levels of seniority within each hospital to ensure a broad starting point. Furthermore, participants were explicitly asked to recommend colleagues with diverse experiences and viewpoints, including those who might have different perspectives on nurse migration. These efforts were combined with maximum variation sampling based on factors such as years of experience as a nurse manager, department (e.g., emergency, medical-surgical), and educational background to capture a range of experiences [45].

### Data collection

Data collection involved face-to-face, semi-structured interviews facilitated by the primary investigator (AG) from June 2024 to September 2024. Prior to the interviews, there was no established relationship between the investigator and the participants, ensuring an unbiased interaction during the data-gathering process. Written informed consent was obtained from all participants before the interviews commenced. The interviews were audio-recorded and conducted in Nepali and English, allowing participants to respond in their preferred language. Both authors are registered nurses and academics in Nepalese healthcare settings who are fluent in Nepali and English. The first author (AG) transcribed all audio-recorded interviews verbatim and translated them into English where necessary. To ensure accuracy, a second author (MSN) independently reviewed a random selection of interview transcripts, including translations. The final transcribed interviews were returned to all participants for validation before the data analysis. The interviews lasted approximately 40–45 min each and were scheduled at the participant's convenience.

Before beginning the interview, the interviewer explained the purpose of the study and provided definitions for key terms, including "brain drain" and "ethical dilemmas." Brain drain was defined as the emigration of skilled healthcare professionals from Nepal to other countries for better opportunities, often resulting in workforce shortages and impacting the quality of care in the home country [46]. Ethical dilemmas were defined as situations where nurse managers faced difficult choices between competing values or obligations [47], particularly in the context of nurse migration and its impact on staffing, patient care, and the overall healthcare system. The distinction between ethical dilemmas and moral distress was also clarified, with moral distress being explained as the psychological distress that arises when one knows the ethically appropriate action to take but is prevented from doing so due to internal or external constraints [44]. Participants were encouraged to ask questions and seek clarification on any terms or concepts before and during the interview. The interview guide (Table 1) outlines the key questions explored during the interviews. These questions were designed to elicit rich descriptions of the participants' experiences and perspectives related to the ethical implications of brain drain.

**Table 1:** Semi-structured interview questions

Question No	Question
1	Can you describe your experiences as a nurse manager in the context of brain drain in Nepal?
2	How has the immigration of nurses affected your workload and the overall functioning of your department/hospital?
3	What ethical dilemmas or challenges have you encountered as a nurse manager due to brain drain?
4	How do you balance your responsibilities to your staff and patients amidst the challenges of brain drain?
5	Have you observed any impact of brain drain on the quality of care provided in your hospital? If so, can you elaborate?
6	In what ways do you think brain drain affects access to healthcare, particularly for vulnerable populations in Nepal?
7	How do you perceive the impact of brain drain on the morale and well-being of the remaining nursing staff?
8	Have you witnessed any instances where brain drain has led to moral distress among yourself or your colleagues?
9	What are your views on the ethical considerations surrounding nurses who choose to emigrate from Nepal?
10	What policy recommendations would you suggest addressing the ethical challenges and systemic consequences of brain drain in Nepal's healthcare system?

### Data analysis

Inductive qualitative content analysis was employed to analyze the interview transcripts [48]. This approach involved systematically coding the data to identify underlying concepts and relationships, ultimately developing themes and subthemes. Open coding was followed by categorization and theme generation. The findings were then interpreted and presented in a report that included direct quotes and descriptions of the phenomenon within the framework of the identified concepts and themes (Table 2). In line with the approach suggested by Rahimi and Khatooni [49], the study aimed for code and thematic saturation rather than data saturation, recognizing participants' diverse experiences and prioritizing the analysis's depth and richness.

**Table 2:** Data analysis process

Meaning Units (Excerpts from Quotes)	Code	Category	Sub-themes	Themes
"fear of making a mistake"- NM2	Fear of Errors	Emotional Impact	Fear of Errors	Moral Distress on the Frontlines
"forced to prioritize, to triage care"- NM8	Triage Decision Burden	Ethical Dilemma	Triage Decision Burdens	Moral Distress on the Frontlines
"Our patients travel for miles, seeking care they can't find…"- NM6	Access to Care Issues	Healthcare Disparity	Rural Healthcare Disparities	Unequal Burden, Unequal Access
"brain drain is like a silent epidemic…"-NM8	Impact on Rural Healthcare	Healthcare Disparity	Rural Healthcare Disparities	Unequal Burden, Unequal Access
"workload shifts… patient safety is compromised"-NM4	Compromised Patient Safety	Systemic Impact	Compromised Patient Safety	The Ripple Effects of Exodus
"losing the next generation of leaders…"-NM5	Hindered Professional Development	Systemic Impact	Hindered Professional Development	The Ripple Effects of Exodus
"Recruiting and retaining nurses has become an uphill battle…"-NM10	Recruitment & Retention Challenges	Systemic Impact	Recruitment & Retention Challenges	The Ripple Effects of Exodus
"It’s a cruel irony. The Nepal government signs deals…"-NM3	Policy Disconnect	Systemic Impact	Recruitment & Retention Challenges	The Ripple Effects of Exodus
"It's bittersweet to see them go…"-NM2	Conflicting Loyalties	Ethical Dilemma	Conflicting Loyalties	Beyond the Hippocratic Oath
"Sometimes I wonder if they feel guilty…"-NM4	Moral Dilemmas of Emigration	Ethical Dilemma	Moral Dilemmas of Emigration	Beyond the Hippocratic Oath
"Brain drain undermines these principles…"-NM7	Need for Policy Interventions	Policy Recommendations	Need for Policy Interventions	Policy at the Crossroads
"We need to build a resilient health system…"-NM6	Investing in the Domestic Healthcare Workforce	Policy Recommendations	Investing in the Domestic Healthcare Workforce	Policy at the Crossroads
"Sustainability is key. We can't rely on foreign aid…"-NM1	Unsustainable Reliance on External Solutions	Policy Recommendations	Addressing Root Causes of Brain Drain	Policy at the Crossroads

### Rigor and trustworthiness

To ensure the trustworthiness of the qualitative data and subsequent analysis, this study adhered to established criteria for rigor, encompassing credibility, dependability, confirmability, and transferability [50].

### Credibility

Credibility, the confidence in the truth of the findings, was established through several strategies. Member checking was a crucial component. After data collection and transcription, an initial thematic analysis, including a summary of the key themes and supporting quotes, was compiled and sent to all 10 participants via email. This summary was presented in both English and Nepali to ensure comprehension. Participants were invited to review the summary and provide feedback on the accuracy and resonance of the themes with their experiences. They were specifically asked to confirm whether the interpretations accurately reflected their intended meanings and if any crucial aspects were missed. Participants were given two weeks to respond, with follow-up reminders sent via phone calls. All participants responded. Feedback received was carefully considered and incorporated into the final analysis.

### Dependability

Dependability refers to the consistency and stability of the findings over time, which was enhanced through a rigorous and transparent research process. The semi-structured interview guide was developed based on a comprehensive literature review on brain drain, nursing workforce challenges, and ethical dilemmas in healthcare. This review involved searching databases such as PubMed, CINAHL, and Scopus using keywords like "brain drain," "nurse migration," "ethical dilemmas," "moral distress," and "healthcare workforce." The search was limited to articles published in English within the last ten years. The identified articles were then screened for relevance to the research question, and those meeting the inclusion criteria were critically appraised to inform the development of the interview guide. The guide was also informed by both authors' insider experience as registered nurses and academics in Nepalese healthcare settings, providing valuable contextual knowledge. Furthermore, the interview guide was reviewed by two specialists: one in nursing education with expertise in workforce issues and another with expertise in qualitative methodology. This expert review ensured the appropriateness and clarity of the interview questions and their alignment with the research question. The detailed documentation of the data collection and analysis processes, including the coding framework and examples of code development, further contributes to the study’s dependability.

### Confirmability

Confirmability, ensuring that the findings are grounded in the data and not influenced by researcher bias, was addressed through several strategies. Reflexivity was a central focus. The research team, comprised of nursing academics and nurses, engaged in ongoing reflection throughout the study. Recognizing that our own experiences as educators and clinicians in the Nepalese context might influence our interpretations, we maintained reflexive journals to document our assumptions, preconceptions, and potential biases. These journals served as a tool for critical self-reflection, allowing us to examine how our perspectives might shape the research process, from data collection to analysis and interpretation. Furthermore, regular bi-weekly team meetings provided a platform for open dialogue and reflexive discussions, where we challenged each other's interpretations and ensured that the emerging themes were grounded in the data rather than our own preconceived notions. For example, during one meeting, the team discussed the potential for their own experiences as nurses in Nepal to influence the interpretation of the theme "Moral Distress on the Frontlines." One researcher initially interpreted the data through the lens of their own experiences of feeling overwhelmed by workload pressures. However, through discussion and a re-examination of the transcripts, the team recognized that the participants' descriptions of moral distress were more closely tied to their inability to provide adequate care due to systemic constraints, such as lack of resources and staff shortages, rather than simply feeling overwhelmed. This led to a refinement of the theme's definition and a more nuanced understanding of the participants' experiences. An audit trail, including detailed transcripts, coding frameworks, and memos documenting analytical decisions, was maintained throughout the study. This audit trail provides evidence of the analytical process and allows for external scrutiny of the findings.

### Transferability

Transferability, pertaining to the potential applicability of the findings to other contexts, was supported through detailed descriptions of the study participants, including their professional roles, years of experience, and the characteristics of the urban and rural hospital settings. This contextual information allows readers to assess the relevance and transferability of the findings to their own settings and situations. While the findings are specific to the experiences of nurse managers in Nepal within the context of brain drain, the identified themes related to moral distress, ethical dilemmas, and the impact on healthcare systems may resonate with similar contexts in other LMICs facing healthcare workforce migration.

### Ethical considerations

This study was approved by the Nepal Health Research Council (approval number- 333/2024) and the institutional review committee (IRC) from Civil Service Hospital, Kathmandu, Nepal, and Mugu District Hospital, Karnali, Nepal. All participants provided written informed consent and were assured confidentiality and anonymity throughout the study. The informed consent process emphasized the voluntary nature of participation, explicitly stating that participants had the right to withdraw from the study at any time without any negative consequences. It was also made clear that there would be no penalty or loss of benefits for refusing to participate or withdrawing from the study. The potential risks and benefits of participation were thoroughly explained. Participants were informed that there were no direct personal benefits anticipated from participating in the study. However, it was emphasized that their participation could contribute to a better understanding of the ethical challenges faced by nurse managers in Nepal due to brain drain. This knowledge could potentially inform policy changes and interventions aimed at improving working conditions and promoting a more sustainable healthcare workforce. The principle of "do no harm" was strictly adhered to throughout the research process. Participants were assured that all information shared would be treated with the utmost confidentiality and that their identities would be protected. All study data, including audio recordings and transcripts, were stored securely on password-protected devices and were only accessible to the research team. Identifying information was removed from the transcripts during the analysis phase to further ensure anonymity. Data will be retained for five years after the completion of the study, after which it will be securely destroyed.

## Results

### Participants’ characteristics

Ten nurse managers (five from an urban hospital and five from a rural hospital) participated in the study. The mean age of the participants was 31, and their average work experience was 7 years. All participants were female, four having a Master’s degree and employed by public hospitals (Table 3). The sample's exclusively female composition reflects the prevailing gender dynamics in Nepal's nursing landscape, where nursing is predominantly perceived as a female-dominated field. This observation is further substantiated by a report from the International Labor Organization indicating that the nursing workforce in Nepal is entirely female [10].

**Table 3:** Socio-demographic characteristics of the participants

Participant	Education	Gender	Hospital (Rural/Urban; Public/Private)	Department	Position	Years of experience
NM1	Bachelor's	Female	Urban; Public	Medical-Surgical	Nurse Manager	5
NM2	Master's	Female	Urban; Public	Pediatrics	Nurse Manager	10
NM3	Bachelor's	Female	Urban; Public	Emergency	Nurse Manager	6
NM4	Bachelor's	Female	Urban; Public	Obstetrics	Nurse Manager	8
NM5	Master's	Female	Urban; Public	Intensive Care	Nurse Manager	7
NM6	Bachelor's	Female	Rural; Public	Emergency	Nurse Manager	7
NM7	Bachelor's	Female	Rural; Public	Medical-Surgical	Nurse Manager	5
NM8	Master's	Female	Rural; Public	Outpatient	Nurse Manager	8
NM9	Bachelor's	Female	Rural; Public	Community Health	Nurse Manager	6
NM10	Master’s	Female	Rural; Public	Emergency	Nurse Manager	9

### Key themes from the data analysis

**Fig. 1:**
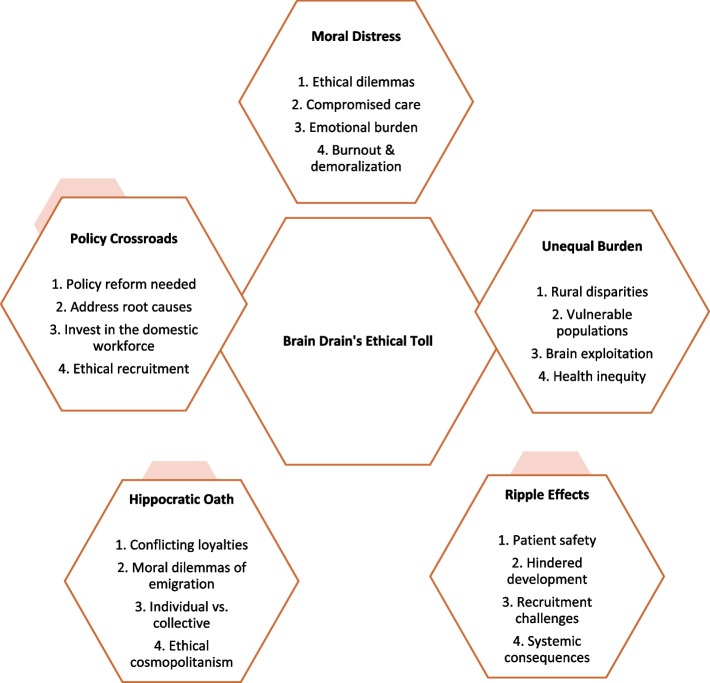
Key themes and subthemes that emerged from the qualitative analysis of interviews with Nepali nurse managers

Thematic analysis of the interview data revealed five core themes that encapsulate the experiences of Nepalese nurse managers in the context of brain drain: (1) Moral Distress on the Frontlines; (2) Unequal Burden, Unequal Access; (3) The Ripple Effects of Exodus; (4) Beyond the Hippocratic Oath; and (5) Policy at the Crossroads. These themes are presented in detail below, with illustrative participant quotes. A comprehensive overview of the data analysis process, including identifying meaning units, codes, categories, and sub-themes, is provided in Table 2 within the Data Analysis section (Fig. 1).

## Findings

### Moral distress on the frontlines

In the heart of Nepal's healthcare system, nurse managers stand as the last line of defense against the relentless tide of brain drain. They frequently find themselves in situations where they must balance their professional obligation to provide optimal patient care with the significant limitations caused by understaffing and a lack of resources. This conflict between their internal moral compass and external constraints creates a breeding ground for moral distress, a state characterized by psychological distress and feelings of powerlessness. As vividly expressed by NM2:

*“Each time a seasoned nurse leaves, it's like losing a limb. We scramble to fill the gaps, stretching ourselves thin, but there's only so much one person can do. The fear of making a mistake, of not being there for a patient in need, it gnaws at you. You question your every decision, wondering if you're doing enough.” (NM2)*

This quote highlights the immediate impact of nurse migration on the workload and the emotional burden placed on those who remain. The fear of making mistakes due to overwork and inadequate staffing is a significant source of moral distress. Nurse managers, particularly in rural settings, often face even more daunting challenges:

*“We're forced to prioritize, to triage care based on limited resources. It's a constant battle between what's ideal and what's possible. Witnessing the desperation in patients' eyes and their families pleading for help, you're torn between your duty to care and the harsh reality of the situation. It's a moral injury that never fully heals.” (NM8)*

This statement underscores the agonizing choices nurse managers must make when resources are scarce, forcing them to compromise ideal care and live with the consequences. The phrase "moral injury" powerfully conveys the lasting psychological impact of these impossible decisions. The cumulative effect of these challenges takes a significant toll, as described by NM7:

*“The weight of responsibility is crushing. "You're not just managing a ward; you're holding together a crumbling system. The constant stress and guilt [...] takes a toll. You try to stay strong for your team and patients, but deep down, you're battling a sense of helplessness. It's a silent struggle that few understand.” (NM7)*

This quote reveals the sense of responsibility nurse managers feel for the overall functioning of the healthcare system, despite its systemic deficiencies. It also highlights the often invisible emotional labor involved in leading during a crisis, further contributing to their moral distress.

### Theme 2: unequal burden, unequal access

The departure of skilled healthcare professionals from Nepal casts a long shadow, particularly over rural communities grappling with limited resources and access to care. The exodus of skilled nurses disproportionately impacts communities already struggling with limited resources, creating an uneven distribution of healthcare services and placing an undue burden on the remaining healthcare workers. As articulated by NM6:

*"We're fighting an uphill battle. Every nurse who departs for the city or overseas leaves a void that's nearly impossible to fill. Our patients travel for miles, seeking care they can't find in their villages. They deserve better, but our hands are tied. It's a constant reminder of the injustice of it all." (NM6)*

This quote underscores the growing gap between healthcare needs and the availability of skilled professionals in rural areas, highlighting the sense of injustice felt by nurse managers who witness the consequences. The impact on rural communities is further emphasized by NM8:

*“The brain drain is like a silent epidemic, eroding the foundations of rural healthcare. We see the consequences every day – delayed diagnoses, untreated complications, preventable deaths. It's a heavy burden to carry, knowing that the most vulnerable among us are paying the highest price.” (NM8)*

This statement paints a grim picture of the deteriorating state of rural healthcare, likening brain drain to a "silent epidemic" that disproportionately affects the most vulnerable populations. It also highlights the emotional toll on nurse managers who witness these preventable tragedies. The strain on the remaining workforce is immense, as expressed by NM10:

*“We try to be everything to everyone, but it's simply not sustainable. We're spread too thin, our resources stretched to the breaking point [….] The mothers who can't reach a hospital in time for childbirth, the elderly left without proper care, the children suffering from treatable diseases... their faces haunt us. It's a moral outrage that demands attention.” (NM10)*

Adding to this complex situation is the financial burden faced by patients:

*“It's heartbreaking to see families forced to choose between food on the table and life-saving treatment. The cost of care has skyrocketed, and many can't afford it. We witness the desperation in their eyes as they plead for help, knowing that without financial assistance, their loved ones may be turned away or forced to abandon treatment altogether. It's a cruel reality that deepens the divide between those who can afford care and those who cannot.” (NM3)*

This statement highlights the intersection of brain drain and socioeconomic disparities, revealing how the lack of access to care is compounded by financial constraints, creating a deeply inequitable system. This theme demonstrates that brain drain is not just about an inadequate workforce but a social justice issue that exacerbates existing inequalities and undermines the fundamental right to healthcare.

### Theme 3: the ripple effects of exodus

This theme captures the cascading consequences of brain drain, extending beyond immediate staffing shortages to impact various aspects of the Nepalese healthcare system. The departure of skilled nurses creates a domino effect, negatively affecting workload, patient safety, mentorship, and the overall sustainability of the workforce. As described by NM4:

*"It's a domino effect," a nurse manager from a busy urban hospital explained. "One nurse leaves, and the workload shifts to those who remain. Fatigue sets in, mistakes become more likely, and patient safety is compromised. We're constantly walking a tightrope, trying to maintain standards while the ground beneath us crumbles.” (NM4)*

The loss of experienced nurses also has long-term consequences for the development of future healthcare professionals, as explained by NM5:

*“We're losing the next generation of leaders. Experienced nurses leave, taking their knowledge and mentorship with them. New graduates are thrown into the deep end, lacking the guidance they need to thrive. It's a vicious cycle perpetuating the brain drain, leaving us an underprepared workforce.” (NM5)*

This statement underscores the crucial role of experienced nurses in mentoring and training new graduates, a role that is severely undermined by their departure. This creates a "vicious cycle" where the lack of mentorship contributes to further brain drain. The challenges are further compounded by systemic issues related to recruitment and retention. NM3 laments:

*“It’s a cruel irony,” a nurse manager in Kathmandu lamented. “The Nepalese government signs deal with countries like the UK, knowing we can’t afford to lose more nurses. And yet, they keep leaving, drawn by promises we can’t match. We're caught in the middle, watching our colleagues disappear, our hospitals hollowed out, and there's little we can do to stop it.” (NM3)*

This quote highlights the complex interplay between national policies and the global demand for nurses, revealing a sense of powerlessness among nurse managers who witness the depletion of their workforce despite government efforts. The difficulties in recruiting and retaining nurses, especially in rural areas, are further emphasized by NM10:

*“Recruiting and retaining nurses has become an uphill battle," a nurse manager from a rural clinic shared. "The allure of better pay and opportunities abroad is hard to compete with. We're forced to make do with less, sometimes compromising on years of experience to keep the lights on. It's a constant ethical dilemma, balancing the need for staff with the imperative to provide safe and effective care.” (NM10)*

This statement underscores the challenges of attracting and keeping qualified nurses in the face of global competition, forcing difficult decisions that can compromise the quality of care. It also highlights the ethical dilemmas inherent in balancing staffing needs with the imperative to provide safe and effective care.

### Theme 4: beyond the hippocratic oath

The decision to leave one's home country to pursue better opportunities abroad is complex and emotionally fraught for Nepali nurses. Their decisions are often filled with conflicting emotions, torn between personal and professional aspirations and a sense of duty to their home country. As observers and confidantes, nurse managers are privy to these internal struggles. NM2 articulates this duality:

*“It's bittersweet to see them go. On the one hand, I'm happy for them, knowing they'll have access to better resources and opportunities. But on the other hand, it's a loss for us and our patients. It's a constant reminder of our systemic challenges, the push and pull factors that drive our brightest minds away.” (NM2)*

The departure of each nurse is perceived as a dual phenomenon: it signifies a celebration of individual career advancement while simultaneously representing a collective loss within the healthcare team. This sentiment reveals the underlying tension between acknowledging the systemic issues that push nurses to leave and the personal desire to retain valuable members of the healthcare team. NM4 offers further insight into the moral weight carried by migrating nurses:

*“Sometimes, I wonder if they feel guilty about leaving behind a system in need. They've dedicated their years to serving their communities and are now walking away. It's a difficult decision, one that weighs heavily on their conscience. You can see the conflict in their eyes, the sadness mixed with hope.” (NM4)*

The moral landscape faced by these nurses is complex. They are not simply abandoning their posts but grappling with a decision that pits their sense of duty against the need for personal and professional advancement. This theme illuminates the often-overlooked emotional and ethical dimensions of brain drain, revealing that the decision to migrate is not merely a career move but a complex moral negotiation for Nepali nurses.

### Theme 5: policy at the crossroads

Acutely aware of the ethical and systemic implications of brain drain, nurse managers advocate for change rooted in equity, sustainability, and moral standards. NM5, from a large urban hospital, challenges the narrative that blames individual nurses for seeking opportunities abroad:

*“We can't simply blame nurses for seeking better opportunities," a nurse manager from a large urban hospital stated. "The WHO's code of ethics emphasizes the right to decent working conditions and fair remuneration. Until we address these systemic issues, the brain drain will persist. We need policies that value our nurses, invest in their growth, and create an environment where they feel respected and supported.” (NM5)*

This perspective shifts the focus from individual choices to the systemic issues that drive brain drain, emphasizing the need for policies that uphold the rights and well-being of healthcare workers. From a rural perspective, NM7 highlights the discrepancy between governmental rhetoric and the reality on the ground:

*“The Nepalese government stresses the importance of nurses' role in promoting health equity and social justice. But it's often limited to words, not actions. Brain drain undermines these principles, leaving vulnerable populations behind. We need policies prioritizing rural healthcare, incentivizing nurses to serve in underserved areas, and ensuring equitable access to care for all.” (NM7)*

This critique exposes the urgent need for concrete policy actions that prioritize rural healthcare and address the widening disparities in access to care, moving beyond mere lip service to tangible change. The need for long-term, sustainable solutions is a common thread, voiced by both urban and rural nurse managers. NM1 cautions against relying on temporary fixes:

*“Sustainability is key. We can't rely on foreign aid and volunteers to solve our problems. They are here temporarily and are gone […]” (NM1)*

This statement serves as a stark reminder of the limitations of short-term solutions and the imperative of building a self-reliant healthcare system. Echoing this sentiment, NM6 calls for a fundamental shift towards a more resilient healthcare system:

*“We need to build a resilient health system, one that can withstand the challenges of brain drain and other crises. This requires long-term investment in education, infrastructure, and the development of a strong and dedicated domestic workforce.” (NM6)*

This vision for a robust healthcare system emphasizes the interconnectedness of education, infrastructure, and workforce development, advocating for sustained investment in these areas to create a system capable of retaining its skilled professionals. This theme underscores a critical juncture for Nepal’s healthcare policy, where decisive action is needed to address the systemic drivers of brain drain and build a future where healthcare is equitable, sustainable, and accessible to all.

## Discussion

The pervasive moral distress experienced by Nepali nurse managers, characterized by the fear of errors, the burden of triage decisions, and the emotional toll of responsibility, serves as a poignant microcosm of a global crisis. The challenges they face resonate deeply with the experiences of healthcare professionals in resource-constrained settings worldwide, where systemic issues such as staff shortages and inadequate resources amplify the risk of moral injury [12]. The emotional toll of these conditions, evident in the nurse managers' narratives of "constant stress," "sleepless nights," and "guilt," underscores the urgent need for interventions that address both the individual and systemic factors contributing to moral distress. While individual resilience can be fostered through self-efficacy, education, and support networks, as demonstrated by frontline nurses during the COVID-19 pandemic [51], sustainable solutions necessitate systemic change. This includes addressing the root causes of brain drain, implementing ethical frameworks, and improving working conditions to create a healthcare environment that supports the well-being of its workforce.

The disproportionate impact of brain drain on Nepal's rural and vulnerable populations mirrors global patterns of health inequity and widening disparities in healthcare access, education, and economic opportunities. Furthermore, the impact is especially pronounced in rural areas, where technological and infrastructural discrepancies further curtail opportunities for those left behind [52]. From a social justice and human rights standpoint, brain drain can be redefined as "brain exploitation” [53], and “reverse foreign aid” [54], underscoring the ethical obligation of high-income countries. These nations benefit from the skills and knowledge of migrants These nations benefit from the skills and knowledge of migrants while often failing to address the negative consequences faced by the source countries. This includes the loss of investment in education and training, the weakening of healthcare systems, and the widening of health inequities [55]. For instance, Nepal invests significant resources in educating and training nurses, only to lose them to high-income nations [56, 57]. This creates a substantial financial and human capital deficit for Nepal, undermining its ability to provide adequate healthcare for its own population [58, 59]. This exploitation is apparent in the migration policies of the Organization for Economic Co-operation and Development (OECD) countries, where support for the reintegration of migrant nurses is lacking [60], and during events like the COVID-19 pandemic when policies were adjusted to "airlift" nurses to address shortages, prioritizing their own immediate needs over the long-term security of the source nations [61]. This perpetuates a cycle of dependency and undermines the principles of global health justice. This calls for a reassessment of migration as a form of exploitation rather than a mere economic opportunity, prompting a critical examination of the power dynamics and structural inequalities perpetuating this phenomenon. The ethical imperative lies in fostering a global healthcare landscape that prioritizes equity, sustainability, and the shared responsibility for ensuring the health and well-being of all populations, regardless of their geographical location or economic status.

Nonetheless, as nurse managers in this study attest, this exodus creates immediate staffing shortages and triggers a cascade of consequences, undermining patient safety and burdening those left behind [62]. Embracing a systems thinking approach [63], is pivotal in comprehending brain drain as a complex adaptive problem. This approach aids in recognizing the interconnectedness of various factors within healthcare systems [64], revealing how the departure of skilled nurses can lead to compromised patient care, decreased morale among remaining staff, and a diminished capacity for training and innovation. Viewing brain drain through this lens allows stakeholders to move beyond addressing immediate staffing shortages and develop multi-faceted adaptive solutions that tackle the root causes of this phenomenon and foster a sustainable healthcare workforce in Nepal.

The ethical dilemmas faced by Nepali nurses who choose to migrate can be explored through ethical cosmopolitanism and the capabilities approach. Ethical cosmopolitanism emphasizes global justice and the moral responsibility to consider the well-being of individuals beyond national borders. This viewpoint helps understand the migration of nurses by acknowledging their right to seek improved living and working conditions [22]. The capabilities approach, which concentrates on enhancing individuals' freedoms and opportunities, further supports the nurses' choices by advocating for their empowerment and pursuing a fulfilling career [65]. The agreement between Nepal and the UK to recruit Nepali nurses initially seemed to represent a step towards ethical cosmopolitanism, promoting fair opportunities and addressing healthcare shortages in the UK while benefiting the migrant nurses and their families [65, 66]. However, this has raised ethical concerns as the UK's nurse recruitment policy now appears to demonstrate little regard for the workforce situations in source countries and the WHO’s ethical recruitment guidelines [67], which prohibit the recruitment of healthcare workers from 'red list' countries such as Nepal that do not have sufficient nurses and doctors for their populations unless there is a government to government agreement in place [68–70]. This inconsistency underscores the complexities of balancing individual rights and global health justice, highlighting the need for ethical migration policies that consider the needs of both sending and receiving countries.

Addressing the complex issue of nurse migration from Nepal requires an interdisciplinary approach that considers the perspectives of health economics, migration studies, and the ethical concerns nurse managers raise. Providing scholarships and financial aid for further education in Nepal could reduce the need for overseas education, often leading to permanent migration [71]. Furthermore, acknowledging the global dynamics of healthcare workforce migration, Nepal could negotiate bilateral agreements with receiving countries, allowing for temporary migration with a guaranteed return, enabling nurses to gain international experience without permanently leaving the domestic workforce [72]. These agreements should also prioritize ethical recruitment practices and ensure provisions for skills transfer back to Nepal. By addressing the root causes of brain drain and prioritizing the needs of nurses and the Nepali healthcare system, policymakers can create a more equitable and resilient healthcare landscape.

## Limitations

The insights presented in this study are derived from the experiences of nurse managers in two hospitals in Nepal, which may limit the generalizability of the findings to other contexts or healthcare settings. The qualitative nature of the study, while offering rich and nuanced data, also carries the potential for subjective interpretation. Additionally, the focus on nurse managers' perspectives leaves room for further exploration of the experiences of other frontline nurses and healthcare professionals impacted by brain drain. Future research could expand the scope of inquiry to include a broader range of healthcare settings and professional roles, contributing to a more comprehensive understanding of the ethical implications of brain drain in Nepal and beyond.

## Conclusion

The exodus of skilled nurses from Nepal, driven by systemic challenges, paints a stark picture of the ethical toll of brain drain. The voices of nurse managers, burdened by moral distress and grappling with the complexities of a depleted workforce, underscore the urgent need for national and global action. Nepal must prioritize comprehensive reforms that address the root causes of brain drain while the HICs benefiting from the influx of skilled healthcare professionals bear an ethical responsibility to collaborate with source countries like Nepal. The findings of this study serve as a clarion call for a paradigm shift in global healthcare workforce policies, one that prioritizes equity, sustainability, and the shared responsibility for ensuring the health and well-being of all populations, regardless of their geographical location.

## Supplementary Information

Supplementary Material 1.

## Data Availability

The data supporting this study's findings are available on request from the corresponding author. However, the data is not publicly available due to privacy or ethical restrictions.
